# Commitments by the biopharmaceutical industry to clinical trial transparency: the evolving environment

**DOI:** 10.1136/bmjebm-2018-111145

**Published:** 2019-03-21

**Authors:** Slavka Baronikova, Jim Purvis, Eric Southam, Julie Beeso, Antonia Panayi, Christopher Winchester

**Affiliations:** 1 Shire International GmbH (now part of Takeda), Zug, Switzerland; 2 Oxford PharmaGenesis Ltd, Tubney, UK

**Keywords:** Clinical trials, transparency, disclosure, reporting

## Abstract

Clinical trial sponsors have ethical obligations to register protocols, report study results and comply with applicable legal requirements. To evaluate public commitments to trial disclosure and rates of disclosure by members and non-members of the European Federation of Pharmaceutical Industries and Associations (EFPIA) and/or the Pharmaceutical Research and Manufacturers of America (PhRMA). Websites of the top 50 biopharmaceutical companies by 2015 sales were searched for statements relating to trial data disclosure. Disclosure of trial results completed by biopharmaceutical industry and non-industry sponsors of at least 30 trials (2006–2015) was assessed using TrialsTracker. Among the top 50 companies, 30 were EFPIA/PhRMA members and 20 were non-members, of which 26 and none, respectively, had a statement on their website committing to the disclosure of trials data. Of 29 377 trials in TrialsTracker, 9511 were industry sponsored (69 companies) and 19 866 were non-industry sponsored (254 institutions). The overall mean disclosure rate was 55%, with higher rates for industry (74%) than for non-industry sponsors (46%). Of the 30 companies within the top 50 with data in TrialsTracker, the mean disclosure rate was 76% (77% for EFPIA/PhRMA members [n=25] vs 67% for non-members [n=5]). Most of the top 50 biopharmaceutical companies have publicly committed to the disclosure of trial data. Industry sponsors have responded to the ethical and legal demands of trial disclosure by disclosing three quarters of their trials compared with less than half for non-industry sponsors. Further improvements in clinical trial disclosure are needed.

## Introduction

A perceived lack of transparency, including under-reporting of results, undermines the confidence of researchers, healthcare professionals and patients in conclusions drawn from clinical trials.^1^ All clinical trial sponsors, be they biopharmaceutical companies or non-industry bodies and triallists, such as government agencies, universities and research charities, have ethical obligations to register applicable trials before they start and to report their results in a timely fashion after they finish.^2 3^ In the USA, EU and elsewhere, it is required that certain types of clinical trial are registered and their results posted on dedicated registries (eg, EudraCT, the EU electronic Register of Post-Authorisation Studies and ClinicalTrials.gov) (online supplementary material, table S1).^4–11^ Other bodies, such as the WHO and the International Committee of Medical Journal Editors (ICMJE), have issued transparency standards and recommendations,^2 12–14^ and some biopharmaceutical companies have websites dedicated to their own trial results.^15 16^ This makes the clinical trial data transparency environment highly complex and diverse.

10.1136/bmjebm-2018-111145.supp1 Supplementary data

10.1136/bmjebm-2018-111145.supp2 Supplementary data

Within the biopharmaceutical industry, which is responsible for approximately half of all clinical trials,^17 18^ two large associations, the European Federation of Pharmaceutical Industries and Associations (EFPIA) and the Pharmaceutical Research and Manufacturers of America (PhRMA), have developed joint ‘Principles for responsible clinical trial data sharing’.^19^ These joint principles, which became effective on 1 January 2014, make the following five commitments:

1. To enhance data sharing with researchers.
2. To enhance public access to clinical study information.
3. To share results with patients who participate in clinical trials.
4. To certify procedures for sharing clinical trial information.
5. To reaffirm commitments to publish clinical trial results.

In the present study, we aimed to evaluate the extent to which EFPIA/PhRMA members and non-members among the leading biopharmaceutical companies have committed to the responsible disclosure of clinical trial results. We also evaluated the reporting of results from clinical trials sponsored by biopharmaceutical companies compared with those from other sponsors.

## Methods

### Commitment to disclosure of clinical trial data by EFPIA/PhRMA member companies

The global public websites of each EFPIA and/or PhRMA (‘EFPIA/PhRMA’) member and non-member company in the top 50 companies by 2015 worldwide prescription sales (‘top 50 companies’)^20^ were searched between December 2017 and January 2018 by one researcher (JP) for direct links to pages containing: (1) a general statement of commitment to disclosing clinical trial data; (2) a general statement of commitment to disclosing clinical trial data according to EFPIA/PhRMA joint principles; and (3) specific statements detailing commitments to upholding one or more of the five individual EFPIA/PhRMA joint principles for responsible disclosure of clinical trial data. If no direct links to such pages were found, the free-text search function of each website was used to search for statements relating to clinical trial data disclosure and implementation of the EFPIA/PhRMA disclosure principles using one or more the key words ‘EFPIA’, ‘PhRMA’, ‘data sharing’, ‘clinical trials’ and ‘transparency’. EFPIA/PhRMA membership was determined from the websites of these two organisations (www.efpia.eu/about-us/membership and http://www.phrma.org/about/members).

Subjective ease of access to relevant information was assessed: good access was rated as requiring either no more than four clicks from the homepage of the company website^21^ or a clear, direct link; poor access was rated as either needing more than four clicks or requiring navigation to satellite websites (eg, blogs).

### Clinical trial results reporting

TrialsTracker is an independent, semiautomated, web-based tool that has been developed in an effort to incentivise sponsors of clinical trials to improve disclosure rates by highlighting the disclosure performance of individual sponsors (trials without results disclosed as a proportion of trials registered).^22^ For clinical trial sponsors to be included in TrialsTracker, they must have more than 30 phase II–IV clinical trials registered on ClinicalTrials.gov that were recorded as completed after 1 January 2006 and at least 24 months before the most recent TrialsTracker update. Because the most recent update to the database was in April 2017, the most recent studies to be included in this analysis were completed in April 2015.

Data detailing the number of trials registered on ClinicalTrials.gov by clinical trial sponsor, and the corresponding number of trials without results reported for each year from 2006 to 2015, were downloaded as a comma-separated values file from the TrialsTracker website (https://trialstracker.ebmdatalab.net). TrialsTracker identifies sponsors as industry (‘biopharmaceutical companies’ which we subcategorised as pharmaceutical/biotechnology, generics/biosimilars, medical devices, plasma products and nutraceuticals, using information on the company websites that was found during the research for our study) or non-industry (classified as National Institutes of Health, US Federal or other) institutions. For each industry and non-industry sponsor and for each category, the number of disclosed trials and the percentage of eligible studies with disclosed results were calculated in Microsoft Excel 2016.

An analysis of disclosure rates was performed on subsets of the industry sponsors within TrialsTracker based on sales revenue (the top 50 companies)^20^ and membership of EFPIA/PhRMA. An arbitrary disclosure rate threshold of 80% was applied to sponsor subgroups.

### Exploratory analyses of results posted on websites other than ClinicalTrials.gov

In an exploratory analysis, clinical trial results from locations other than ClinicalTrials.gov or from linked publications in PubMed were sought for three studies that were selected from four of the top 50 companies. ClinicalTrials.gov was searched by National Clinical Trial (NCT) identifier in order to establish the presence or absence of posted results and/or links to publications on PubMed. We made a separate search of PubMed, Google Scholar and Google using the NCT identifier and a search of EudraCT and the relevant company’s website based on NCT identifier and study title.

### Exploratory analysis of commitments to disclosure of clinical trial data by non-industry sponsors

In an exploratory analysis, we searched for statements relating to the disclosure of clinical trial data on the websites of 10 non-industry sponsors of clinical trials with results completed in the period 2006–2015.

### Data analysis

Disclosure rates for all industry sponsors, the top 50 companies and EFPIA/PhRMA members in the top 50 companies were compared with those for non-industry sponsors.

### Patient involvement

Patients were not directly involved in conducting this study, but patients’ perspectives were sought during the development of the manuscript.

## Results

### Commitment to disclosure of clinical trial data

#### EFPIA/PhRMA membership

Of the top 50 companies, 6 were EFPIA members only, two were PhRMA members only, 22 were both EFPIA and PhRMA members and 20 were neither EFPIA nor PhRMA members. There were more EFPIA/PhRMA members in the largest 25 companies (n=23) than in the second group of 25 largest companies (n=7) (online supplementary material, table S2).

All 30 EFPIA/PhRMA members in the top 50 companies were pharmaceutical/biotechnology companies, whereas the 20 non-members were more varied, comprising pharmaceutical/biotechnology (n=8), generics/biosimilars (n=6), medical devices (n=1), both generics and medical devices (n=2), intravenous products and medical devices (n=1), plasma products (n=1) and nutraceuticals (n=1) companies. Of the two EFPIA/PhRMA non-members in the top 25 companies, one was a biotechnology company and the other was a generics company.

#### Access to a general disclosure statement

A general statement committing to the disclosure of clinical trial information was found on 26 of the top 50 company websites (52%), all of which were EFPIA/PhRMA members (table 1). In 19 cases (38% of the top 50 companies; 63% of EFPIA/PhRMA members), the statement was found within four clicks of entering the website; in seven cases, access was rated as poor. An analysis of the proportion of EFPIA/PhRMA members versus non-members in the top 50 companies with statements committing to responsible data transparency is shown in online supplementary material, table S3.

**Table 1:** Number of EFPIA and PhRMA member and non-member companies in the top 50 biopharmaceutical companies as ranked by 2015 worldwide prescription sales and their public commitment to disclosing clinical trial data

Top 50 companies: membership of EFPIA and/or PhRMA	General data sharing statement, n (%)	EFPIA/PhRMA principles	Five EFPIA/PhRMA joint principles of responsible clinical data sharing						
			Commitment to sharing clinical trial data with researchers	Public availability of CSR synopsis as a minimum	Availability of results for trial participants	Public certification of adoption of EFPIA/PhRMA commitments	Commitment to publish clinical trial data (phase III minimum)	All five principles	
Member (n=30)	26 (86.7)	20 (66.7)	25 (83.3)	22 (73.3)	18 (60.0)	21 (70.0)	24 (80.0)	16 (53.3)	
Non-member (n=20)	0 (0.0)	0 (0.0)	0 (0.0)	1 (5.0)	0 (0.0)	0 (0.0)	0 (0.0)	0 (0.0)	
Total (n=50)	26 (52.0)	20 (40.0)	25 (50.0)	23 (46.0)	18 (36.0)	21 (42.0)	24 (48.0)	16 (32.0)	

CSR, clinical study report; EFPIA, European Federation of Pharmaceutical Industries and Associations; PhRMA, Pharmaceutical Research and Manufacturers of America.

#### Specific EFPIA/PhRMA principles

An overview statement referring to the adoption of the joint principles (http://phrma-docs.phrma.org/sites/default/files/pdf/PhRMAPrinciplesForResponsibleClinicalTrialDataSharing.pdf) was found on 20/30 websites (67%) of EFPIA/PhRMA members. Reference to all five joint principles was found for 16/30 members (53%). Of non-member companies, only one company made a specific disclosure statement (to enhance public access to clinical study information by making synopses of clinical study reports publicly available) (table 1). The most frequently communicated individual commitments were to share clinical trial data with researchers (83% of EFPIA/PhRMA members; 50% of the top 50 companies) and to publish clinical trial data (80% of members; 48% of the top 50 companies).

### Clinical trial results reporting

**Figure 1:**
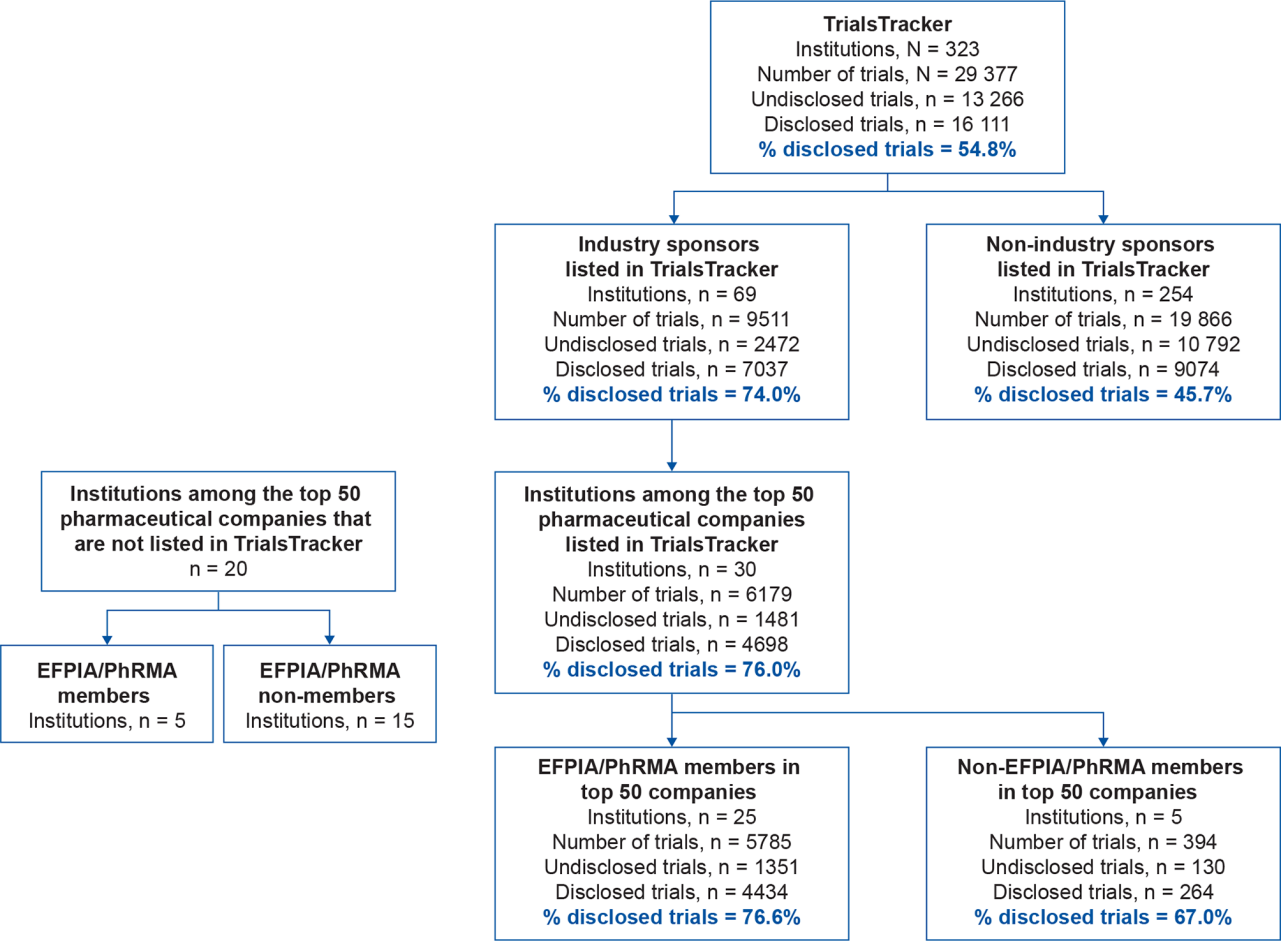
Types and numbers of sponsors represented in TrialsTracker, with their disclosure rates. EFPIA, European Federation of Pharmaceutical Industries and Associations; PhRMA, Pharmaceutical Research and Manufacturers of America.

**Figure 2:**
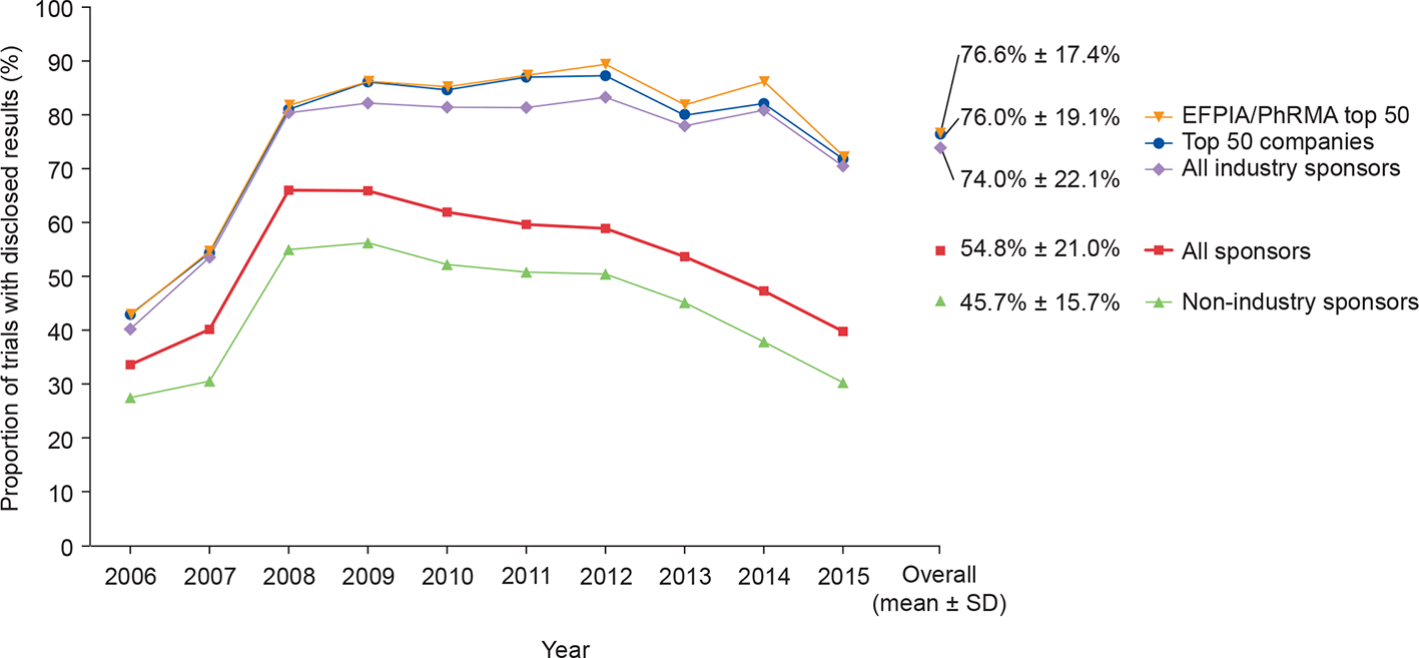
Disclosure of clinical trial results by sponsor type. EFPIA, European Federation of Pharmaceutical Industries and Associations; PhRMA, Pharmaceutical Research and Manufacturers of America.

Of 29 377 trials listed in TrialsTracker, 9511 (32%) were sponsored by 69 biopharmaceutical companies (a mean of 138 trials per company) and 19 866 (68%) were sponsored by 254 non-industry institutions (a mean of 78 trials per institution) (figure 1). Of all 13 266 undisclosed trials, 10 792 (81%) were sponsored by non-industry institutions and 2474 (19%) were sponsored by industry. The mean±SD disclosure rate for all trials was 55%±21.0%, with higher rates for industry (7037/9511; 74%±22.1%) than for non-industry sponsors (9074/19 866; 46%±15.7%) (figure 2; online supplementary material, table S3).

The overall disclosure rate for all clinical trials substantially increased during 2007 and 2008 before declining thereafter. The maximum mean disclosure rate for all clinical trials was observed in 2008 (2069/3135; 66%); for industry-sponsored and non-industry-sponsored trials, the maximum mean disclosure rates were in 2012 (761/914; 83%) and 2009 (1160/2063; 56%), respectively (figure 2). Disclosure rates for non-industry sponsors declined after 2009, whereas disclosure rates for industry sponsors were maintained at approximately 80% until 2014 (figure 2).

**Figure 3:**
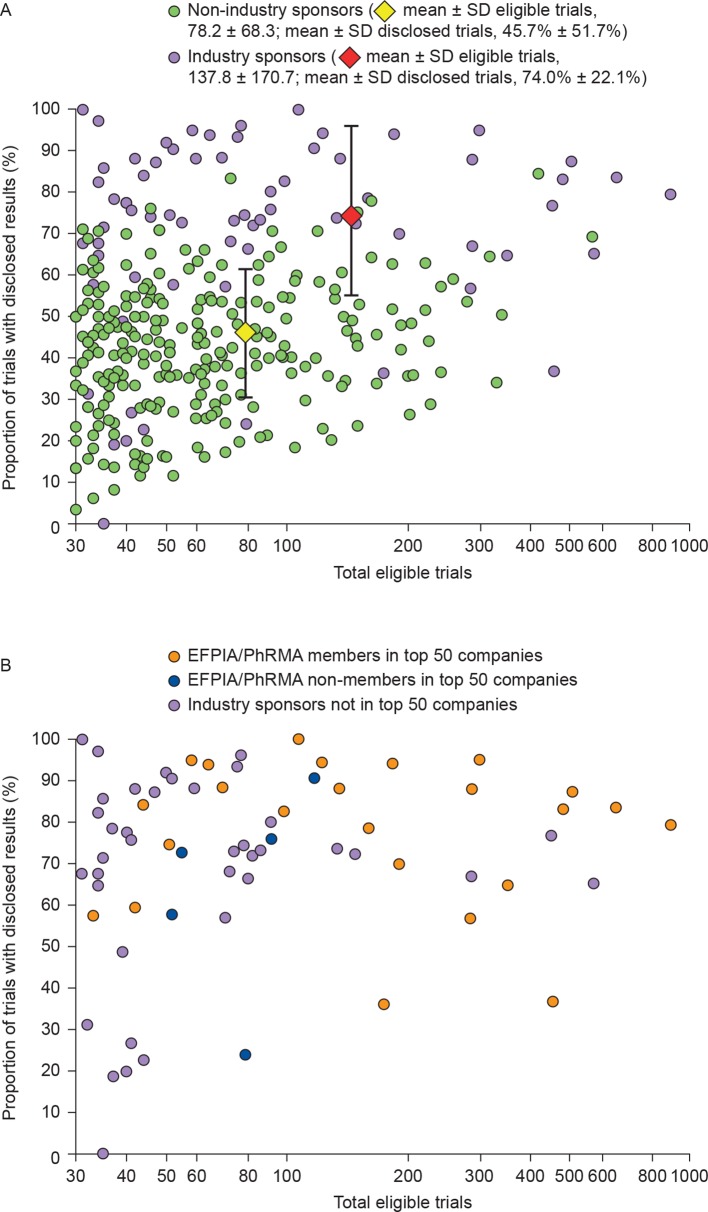
Disclosure rates versus total eligible trials for (A) biopharmaceutical industry and non-industry clinical trial sponsors and (B) industry-only sponsors, highlighting EFPIA/PhRMA members and non-members in the top 50 companies, and industry sponsors not in the top 50 companies. Whiskers represent SD. EFPIA, European Federation of Pharmaceutical Industries and Associations; PhRMA, Pharmaceutical Research and Manufacturers of America.

There was high variability in disclosure rate between sponsor type (figure 3A). The highest disclosure rate achieved by a non-industry sponsor was 353/418 (84%), whereas two biopharmaceutical industry sponsors achieved 100% disclosure. Of the top 50 companies, a mean±SD of 76%±19.1% of trials were disclosed by the 30 companies with data reported in TrialsTracker (all of which were pharmaceutical/biotechnology companies). The mean±SD disclosure rate was 77%±17.4% for EFPIA/PhRMA members (4434/5785 trials; 25 companies) and was 67%±25.3% for non-members (264/394; 5 companies) (figures 2 and 3B). An arbitrary disclosure rate threshold of 80% was reached by fewer than 1% (2/254) of non-industry sponsors compared with 39% (27/69) of industry sponsors. Of the 69 biopharmaceutical industry sponsors with results in TrialsTracker, the 80% threshold was met by 57% (13/23) of EFPIA/PhRMA members in the top 50 companies, by 20% (1/5) of EFPIA/PhRMA non-members in the top 50 companies and by 31% (12/39) of biopharmaceutical industry sponsors that were not in the top 50 companies (figure 3B).

### Exploratory analyses

In the first exploratory analysis, commitments to disclosure of clinical trial data by non-industry sponsors were made by 1/10 institutions (online supplementary material, table S4). In the second exploratory analysis, clinical trial results from four industry sponsors that were not posted on ClinicalTrials.gov were disclosed on a variety of other websites (eg, EU Clinical Trials Register and company website) as summarised in online supplementary material, table S5.

## Discussion

This analysis of the disclosure environment of clinical trial sponsors began with a review of the publicly stated disclosure policies of the top 50 biopharmaceutical companies. Of these, 26 companies (52%; all of which were members of one or both of the two leading international industry bodies [EFPIA and PhRMA]) made their disclosure policies easily available on their websites. Most EFPIA/PhRMA members (87%) communicated that they had a commitment to disclose clinical trial results and two-thirds (67%) specifically referred to the EFPIA/PhRMA joint principles; approximately half (53%) described those principles in detail.

To be useful, information on websites should be easy to find and have a logical flow. Research has shown that four clicks may be viewed as the minimum required to achieve any level of success for completing a search^21^; the ‘three-click rule’ is no longer regarded as the benchmark for website utility.^21 23^ Inherent in the principle of publicly committing to data disclosure should be that the statements have good accessibility.

The second phase of our study collated disclosure information from a large number of trials and sponsors over a 10-year period using data from TrialsTracker. This showed that the disclosure of clinical trial data remains suboptimal. By the end of April 2017, data were disclosed for approximately half of the phase II–IV trials registered on ClinicalTrials.gov and completed in the period 2006–2015. Over this period, results were disclosed by approximately three-quarters of the biopharmaceutical industry sponsors compared with less than half of the non-industry sponsors. Of undisclosed trials, more than 80% were originally funded by governmental, charitable or academic institutions compared with just under 20% by industry, even though approximately one-third of all trials in our data set were industry funded. Disclosure rates for both types of funder substantially increased between 2007 and 2008, coinciding with mandatory reporting as required by the Food and Drug Administration Amendments Act 801 (FDAAA 801). For industry sponsors, disclosure rates were maintained for the next 6 years before declining slightly in 2015. This decline may reflect delays in the publication process, which usually takes approximately 2 years from study completion.^24^ With the implementation of the ‘Final Rule’ in January 2017, there should no longer be delays in the posting of results from applicable clinical trials on ClinicalTrials.gov.^24–26^ By contrast, disclosure rates for non-industry studies steadily declined after 2009, possibly reflecting a lack of policing of FDAAA 801. It is noteworthy that those companies within the top 50, which as members of EFPIA/PhRMA, have committed to adhere to the EFPIA/PhRMA principles,^27^ have numerically the highest mean disclosure rate.

The proportion of trials identified in the present study as sponsored by the biopharmaceutical industry (approximately one-third of all trials) was similar to that reported previously.^17 26^ In our analysis, the disclosure rate for industry-sponsored studies was similar to that previously observed using TrialsTracker and the EU Clinical Trials Register (EUCTR),^22 28^ although this rate is lower than the rates reported for newly approved drugs in the USA and Europe,^29 30^ and either lower than or similar to rates of publication that have been reported by single sponsors,^24 25^ and with a similar study design profile to a previously reported study.^18^ Similarly, for non-industry studies, the disclosure rate was similar to that previously seen with TrialsTracker,^22^ and either lower than or similar to those reported for academic medical centres in the USA and the UK but higher than for EUCTR.^18 28 31 32^

Our assessment of biopharmaceutical company disclosure policies showed results similar to those from a recent EFPIA/PhRMA survey in which 77% of the 44 EFPIA/PhRMA members confirmed that they state on a publicly available website that they adhere to the joint principles.^27^ In a recent survey of the internal disclosure policies of 25 top biopharmaceutical companies, 96% reported that they had a policy committing to the sharing of summary results in academic articles or on a clinical trial registry.^33^ However, in the present study, we found such commitments on the websites of only about half of the top 50 companies, suggesting that many companies are missing the opportunity to inform the general public about their disclosure policies.

In contrast to the results for EFPIA/PhRMA members, but in line with those for non-member companies, a preliminary review of commitments to data transparency that was conducted for 10 non-industry sponsors of clinical trials completed in the period 2006–2015 demonstrated that only one institution referred to the disclosure of clinical trial data (online supplementary material, table S4). The requirement for EFPIA/PhRMA members to commit publicly to data disclosure is reflected in clear differences between these companies and non-members/non-industry sponsors. A summary of the findings from this study is presented in an infographic that can be found in online supplementary material.

Because we used data from TrialsTracker, which searches only on ClinicalTrials.gov, the largest available clinical trial registry, using only NCT identifiers, we made an exploratory search of alternative sources of clinical trial data for 12 clinical trials sponsored by four of the top 50 biopharmaceutical companies; although these analyses were not performed with the same rigour or level of detail as the main analyses, they show that some results that were missing from ClinicalTrials.gov were found on EudraCT and company websites, suggesting that TrialsTracker was underestimating the number of trials that had published results. As recommended by the ICMJE, the inclusion of the study, NCT and/or EudraCT numbers in the abstract of publications linked to clinical trials would help to improve assessments of the disclosure of clinical trial data.^7^

Conducting clinical trials requires a high level of trust between the patient, the medical team and the trial sponsor. Central to the trust placed in the sponsor by the patient is that the results of the trial will be made publicly available. Many patients enrol in clinical trials in the hope of improving their own health and in the expectation that their participation will contribute to a better understanding of their condition and to the development of potential new treatments. Participants must weigh these potential benefits against the risk of adverse reactions. For their involvement in clinical trials to have meaning to the participants, all trial sponsors should transparently report all results, both positive and negative.

### Strengths and limitations

Our study was based on an evaluation of a large number of phase II–IV clinical trials from industry and non-industry sponsors over a 10-year period; however, several caveats should be considered when interpreting the results. First, it should be noted that the results obtained from the automated data acquisition system used by TrialsTracker are subject to error in the reporting rate. In a previous article,^22^ Powell-Smith and Goldacre compared their results in TrialsTracker with those from a previous manual audit of the disclosure of results from 4347 trials (performed by Chen *et al*).^31^ Of the 2562 trials in both analyses, 1149 were found to be reported in both, 534 were unreported by both, 497 were reported by Chen *et al* but not by Powell-Smith and Goldacre and 382 were unreported by Chen *et al* but were reported by Powell-Smith and Goldacre. Thus, the total number of discordant trials (874 of 2562) represents 34.3% of the trials in both analyses. However, when analysing the results from Powell-Smith and Goldacre in comparison with those from Chen *et al* more closely, 14.9% of the trials were ‘overreported’ and 19.4% were ‘underreported’, which may be interpreted as a net underestimation of the reporting rate by 4.5%; underestimation of studies performed by industry sponsors may be a particular issue because many companies only disclosed their results on their own websites. Second, only two types of disclosure were included: publication in a journal and posting of results on ClinicalTrials.gov. Because publications were identified through automated searches of PubMed for NCT identifiers, identification and discoverability were limited to trials published with NCT identifiers included in the secondary source ID field of PubMed, title or abstract.^34 35^ Results disclosed elsewhere (eg, institutional websites and other registries) or published without reference to the NCT identifier could lead to the understating of disclosure rates. Third, we may not be able to generalise our findings to all sponsors and clinical trials because our analysis included sponsors of only 30 or more trials (CCW has previously calculated that sponsors of 30 studies or fewer are responsible for approximately half of all registered trials).^36^ Fourth, our study looked only at the disclosure of registered studies but not all studies are registered; indeed, unregistered studies seem to be less likely than registered studies to be published.^37^ Finally, our analysis of publicly available disclosure policies used key word searches that focused on disclosure, so it is possible that specific publication policies were missed. Nevertheless, our findings suggest that statements related to the disclosure of results are difficult to find in many cases.

The problem of incomplete and inconsistent clinical trial disclosure remains despite public awareness campaigns and the introduction of various policies, legislation and fines. Company-sponsored trials have been the focus of many of these activities because of their perceived commercial influence. However, the present data demonstrate that results from trials sponsored by the biopharmaceutical industry are disclosed more often than those from non-industry funded studies. The results of our analysis agree with those from two recent studies that reported that industry funders disclose the results from a higher proportion of their trials than do non-industry funders.^18 28^ These findings may reflect the considerable resources that commercial organisations have dedicated to clinical trial disclosure. They also suggest that the focus of future efforts to improve trial disclosure should shift towards the development of a single set of globally applicable rules and harmonisation of clinical trial data transparency principles to make them more easily implemented by organisations without the resources of pharma companies. This could be achieved by active discussion between, and endorsement by, all stakeholders, including clinical trial sponsors, regulatory bodies and other public bodies (eg, WHO, ICMJE and EU Council), as well as those campaigning for increased transparency of clinical trial information. We recommend that investigators do not begin recruitment and patients do not participate in clinical trials unless they have been registered on an international registry. We believe that well-defined EFPIA/PhRMA joint principles could be used as a basis for the development of harmonised transparency and disclosure principles and meanwhile should be established as an example of best practice in order to encourage consensus. Simplification of the transparency rules and regulations, the implementation of a single study identifier that can be used across all registries, publications and results databases and improved scrutiny of compliance should extend across all aspects of clinical trials and sponsors.

**Summary box**

**What is already known on this subject?**

- Clinical trial sponsors are ethically and legally bound to register every trial and report the results in a timely fashion.
- Not all clinical trials are reported via public registries and/or primary manuscripts.
- By joining the European Federation of Pharmaceutical Industries and Associations (EFPIA) and/or the Pharmaceutical Research and Manufacturers of America (PhRMA), biopharmaceutical companies make a commitment to reporting clinical trial data following EFPIA/PhRMA joint principles for responsible data sharing.

**What are the new findings?**

- Publicly stated commitment to transparency in clinical trial data sharing and disclosure is more common among biopharmaceutical companies that are members of EFPIA and/or PhRMA than among non-members.
- Disclosure rates for studies registered on ClinicalTrials.gov for biopharmaceutical industry sponsors are higher than those for non-industry sponsors, which are declining.
- Preliminary data suggest that a reliance on studies registered on ClinicalTrials.gov and publications that quote the NCT number may lead to underestimation of clinical trial disclosure rates.
- Most undisclosed clinical trials are sponsored by non-commercial organisations such as government agencies, research charities and universities rather than by the biopharmaceutical industry.

**How might these results change the focus of research or clinical practice?**

- Further improving of clinical trial transparency and data disclosure may be achieved through simplification of the transparency rules and regulations, the implementation of a single study identifier that can be used across all registries, publications and other results databases and improving the scrutiny of compliance across all aspects of clinical trials.
