# Supplementary material for: Commitments by the biopharmaceutical industry to clinical trial transparency: the evolving environment
Source: BMJ Evid Based Med. 2019 Mar 21;24(5):177–84. doi: 10.1136/bmjebm-2018-111145 (PMC6837245; doi:10.1136/bmjebm-2018-111145)
Supplement: Supplementary data [file bmjebm-2018-111145supp001.pdf]

Transparency in clinical research

Disclosing clinical trial data: EFPIA and PhRMA

- All sponsors of clinical research have an ethical obligation to register protocols and disclose the results of clinical trials in a timely and transparent manner*
- Industry sponsors can become members of the trade organisations EFPIA and/or PhRMA and subscribe to their joint principles for responsible disclosure of clinical trial data:
  - To enhance data sharing with researchers
  - To enhance public access to clinical study information
  - To share results with patients who participate in clinical trials
  - To certify procedures for disclosing clinical trial information
  - To reaffirm commitments to publish clinical trial results

*Clinical trial sponsors must comply with the legal requirements imposed by regulators (including, but not limited to, FDAAA 801 'The Final Rule' and the European Medicines Agency clinical trial regulation EU number 536/2014)

European Federation of Pharmaceutical Industries and Associations and Pharmaceutical Research and Manufacturers of America. EFPIA and PhRMA joint principles for responsible clinical trial data sharing. 2013. Available from **http://phrma-docs.phrma.org/sites/default/files/pdf/PhRMAPrinciplesForResponsibleClinicalTrialDataSharing.pdf**. Accessed 12 February 2018.

Methods

What proportion of all included trials was disclosed by sponsors?

- Trial disclosure commitments were searched for on the public website of each EFPIA/PhRMA member and non-member in the top 50 companies
- The web-based TrialsTracker tool‡ identified sponsors with more than 30 phase 2–4 clinical trials on ClinicalTrials.gov and disclosed data there or in scientific publications (PubMed)
  - Industry sponsors: pharmaceutical, biotechnology, generics/biosimilars and devices
  - Non-industry sponsors: NIH, US federal governments or other

74%

69 industry sponsors: 9511 trials

46%

254 non-industry sponsors: 19 866 trials

‡Available at **https://trialstracker.ebmdatalab.net/#/**

Do industry sponsors commit to the EFPIA/PhRMA principles?

Looking at the top 50 companies†…

26/30 are committed to data sharing

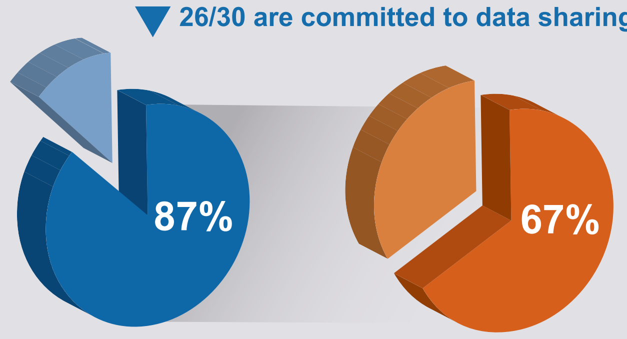

20/30 committed to EFPIA/PhRMA principles, and 16 of these 20 committed to all five principles

Of 20 non-members, none committed to sharing data from clinical trials or to all five principles

†As ranked by 2015 worldwide prescription sales

Do sponsors meet the requirement to publish their results?

Industry sponsors disclosed 80% of trials in 2008

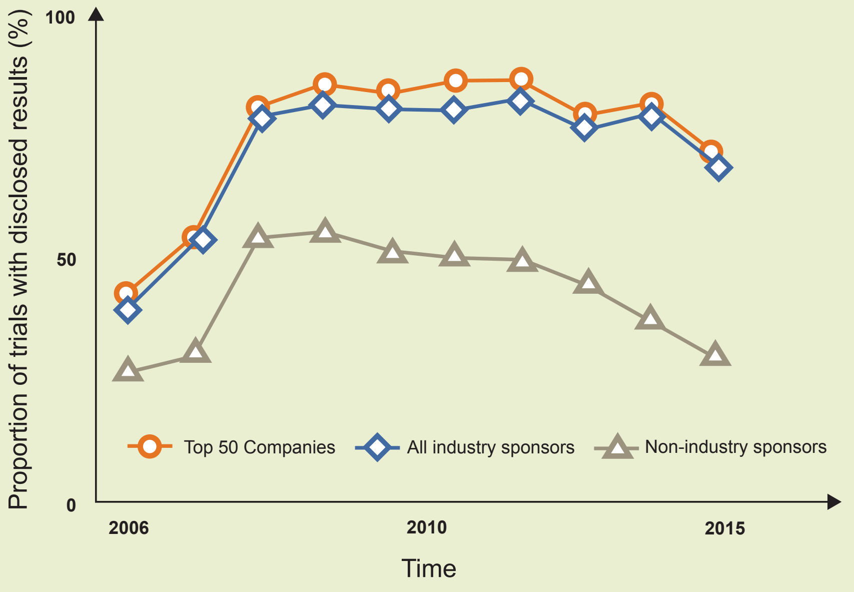

Industry sponsors share a bigger proportion of results from their trials than non-industry sponsors

Who met an 80% disclosure rate threshold?**

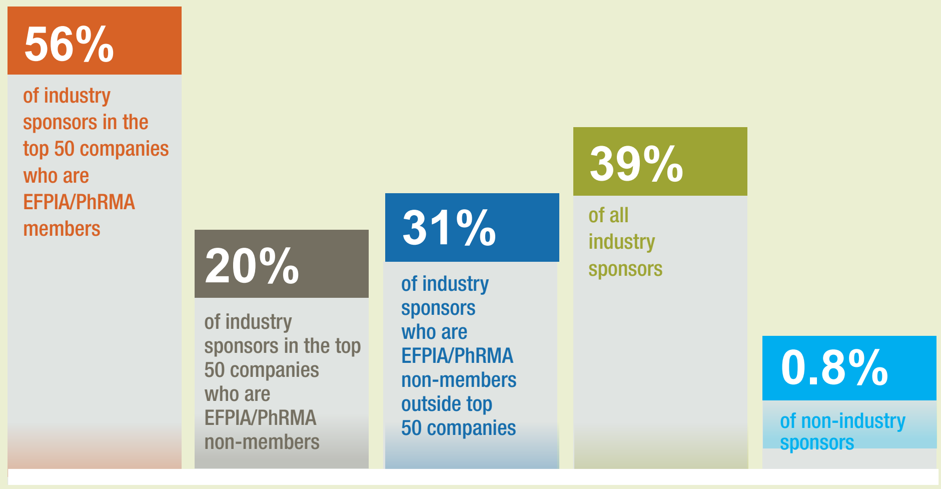

100% disclosure rate from two industry sponsors

Maximum 84% disclosure rate from a non-industry sponsor

**arbitary disclosure rate

Key findings

- **Public commitment to transparency in clinical trial disclosure on company websites is higher among pharmaceutical companies that are members of EFPIA and/or PhRMA than among non-members**
- **Overall, disclosure rates for industry sponsors are higher than those for non-industry sponsors**
- **Disclosure rates are not optimal and could be raised**

Acronyms: EFPIA, *European Federation of Pharmaceutical Industries and Associations*; NIH, *National Institutes of Health*; PhRMA, *Pharmaceutical Research and Manufacturers of America*
