# Supplementary material for: Commitments by the biopharmaceutical industry to clinical trial transparency: the evolving environment
Source: BMJ Evid Based Med. 2019 Mar 21;24(5):177–84. doi: 10.1136/bmjebm-2018-111145 (PMC6837245; doi:10.1136/bmjebm-2018-111145)
Supplement: Supplementary data [file bmjebm-2018-111145supp002.pdf]

Commitments by the biopharmaceutical industry to clinical trial transparency: the evolving environment

Supplementary material

Table S1 Key elements of the ‘Final Rule’, EMA clinical data publication policy 0070 and EMA clinical trial regulation EU no. 536/2014

	**FDAAA 801 the ‘Final Rule’[1]**	**EMA clinical data publication policy 0070[2]**	**EMA clinical trial regulation EU no. 536/2014[3 4]**
**Medicinal products covered**	FDA-regulated products that have not yet been approved, licensed or cleared by the FDA	Centrally authorised products only	Investigational medicinal products regardless of whether they have a marketing authorisation
**Clinical studies covered**	Includes all clinical trials for products as listed above	Clinical studies submitted to the agency as an MAA, Article 58, line extension or new indication, regardless of where the study was conducted	Clinical trials conducted in the EU and paediatric trials conducted outside the EU that are part of paediatric investigation plans
**Documents published**	Additional registration, summary results, full protocol and SAP, including more frequent updates to posted data	Clinical data overview (clinical overview, clinical summaries and CSRs) and the anonymisation report	All clinical trial-related information generated during the life cycle of a clinical trial (e.g. protocol, assessment of and decision on trial conduct, and summary of trial results including a lay summary, study reports and inspections)
**Publication channel**	ClinicalTrials.gov	https://clinicaldata.ema.europa.eu	Future EU portal and database (https://eudract.ema.europa.eu until that time)
**Effective date**	18 January 2017	1 January 2015 (MAA or Article 58) or 1 July 2015 (line extension or new indication)	October 2018
**Posted from**	18 April 2018	October 2016	2019

Adapted from: Panayi A, Baronikova S. A new age of transparency: do we fully understand the implications? International Society for Medical Publication Professionals, Inc. (ISMPP). 2017.

CSR, clinical study report; EMA, European Medicines Agency; FDA, US Food and Drug Administration; FDAAA, US Food and Drug Administration Amendments Act; MAA, marketing authorisation application; SAP, statistical analysis plan.

Table S2 EFPIA and/or PhRMA membership status and availability of results in TrialsTracker (December 2017–January 2018)

Company rank by 2015 worldwide prescription sales*	EFPIA only†	PhRMA only†	Both EFPIA & PhRMA†	Not EFPIA or PhRMA†	Results in TrialsTracker‡
Pfizer			✓		✓
Novartis			✓		✓
Roche	✓				X
Merck (Merck.com)			✓		✓
Sanofi			✓		✓
Gilead Sciences				✓	✓
J & J			✓		✓
GSK			✓		✓
AstraZeneca			✓		✓
Abbvie			✓		✓
Amgen			✓		✓
Allergan		✓			✓
Teva			✓		X
Novo Nordisk			✓		✓
Eli Lily			✓		✓
Bayer			✓		✓
Bristol-Myers Squibb			✓		✓
Takeda			✓		✓
Boehringer Ingelheim			✓		✓
Astellas Pharma			✓		✓
Mylan				✓	X
Biogen			✓		✓
Celgene			✓		✓
Merck KGaA (Merckgroup.com)	✓				✓
Daiichi Sankyo			✓		✓
Valeant Pharma Int				✓	✓
Otsuka			✓		✓
CSL				✓	X
Baxalta				✓	X
Shire	✓				✓
Sun Pharma Ind				✓	X
Servier	✓				X
Eisai			✓		✓
UCB	✓				✓
Abbott Labs				✓	✓
Fresenius				✓	X

Grifols				✓	X
Chugai Pharma				✓	X
CJ (CheilJedand)				✓	X
Malincrodt				✓	X
Sumitomo				✓	X
Endo Int				✓	X
Menarini	✓				X
Regeneron Pharmaceuticals				✓	X
Alexion Pharma		✓			X
Aspen Pharmacare				✓	X
Mitsubishi Tanabe Pharma				✓	✓
Nestlé				✓	✓
Meda				✓	X
Hospira				✓	X

*EvaluatePharma®, April 2016.

†EFPIA/PhRMA members webpage, December 2017–January 2018.

‡TrialsTracker (updated April 2017).

Data analysis

A comparison of proportions (e.g. all industry, the top 50 companies and EFPIA/PhRMA members in the top 50 companies compared with non-industry clinical trial sponsors, and EFPIA/PhRMA members vs non-members in the top 50 biopharmaceutical companies with statements committing to responsible data transparency) was calculated as follows (note that the comparison of clinical trial disclosure for industry and non-industry sponsors is used as an example):

- proportion of trials disclosed by industry (p^1) = $\frac{\text{disclosed industry trials } (n^1)}{\text{total industry trials } (N^1)}$
- proportion of trials disclosed by non-industry (p^2) = $\frac{\text{disclosed non-industry trials } (n^2)}{\text{total non-industry trials } (N^2)}$
- sample proportion ($\hat{p}$) = $\frac{((p^1 \times N^1)+(p^2 \times N^2))}{(N^1+N^2)}$
- standard error (SE) = $\sqrt{((\hat{p} \times (1-\hat{p})) \times \frac{(N^1+N^2)}{(N^1 \times N^2)})}$
- *z*-score = $\frac{(p^1 - p^2)}{SE}$

Confidence level	***z*-score (standard deviations)**	P value
90%	< –1.65 or > +1.65	< 0.10
95%	< –1.96 or > +1.96	< 0.05
99%	< –2.58 or > +2.58	< 0.01

- 95% confidence interval for the difference in the proportion of disclosed clinical trials ($p^1 - p^2$):
  - lower limit = $(p^1 - p^2) - (1.96 \times SE)$
  - upper limit = $(p^1 - p^2) + (1.96 \times SE)$

The null hypothesis (H_0) was for no difference in the proportions of disclosed trials between industry and non-industry sponsors.

Table S3 Comparison of disclosure rates for all industry, the top 50 biopharmaceutical companies and EFPIA/PhRMA members in the top 50 biopharmaceutical companies compared with non-industry sponsors.

Year	Sponsor	N	n	Proportions, %	SE	95% CI	Significance
**2006**	All industry	851	342	40.2	0.022	0.083–0.171	$p < 0.01$
	Non-industry	921	253	27.5			
**2007**	All industry	1063	569	53.5	0.020	0.192–0.269	$p < 0.01$
	Non-industry	1478	451	30.5			
**2008**	All industry	1361	1094	80.4	0.017	0.221–0.288	$p < 0.01$
	Non-industry	1774	975	55.0			
**2009**	All industry	1223	1005	82.2	0.017	0.226–0.293	$p < 0.01$
	Non-industry	2063	1160	56.2			
**2010**	All industry	1108	902	81.4	0.018	0.257–0.327	$p < 0.01$
	Non-industry	2217	1157	52.2			
**2011**	All industry	997	811	81.3	0.018	0.270–0.342	$p < 0.01$
	Non-industry	2444	1241	50.8			
**2012**	All industry	914	761	83.3	0.019	0.291–0.365	$p < 0.01$
	Non-industry	2630	1326	50.4			
**2013**	All industry	916	714	77.9	0.019	0.291–0.366	$p < 0.01$
	Non-industry	2609	1177	45.1			
**2014**	All industry	763	617	80.9	0.020	0.390–0.470	$p < 0.01$
	Non-industry	2712	1026	37.8			
**2015**	All industry	315	222	70.5	0.032	0.340–0.464	$p < 0.01$
	Non-industry	1018	308	30.3			
**Overall**	All industry	9511	7037	74.0	0.006	0.271–0.295	$p < 0.01$

Non-industry	19 866	9074	45.7			
Top 50	6179	4698	76.0	0.007	0.289–0.318	$p < 0.01$
Non-industry	19 866	9074	45.7			
EFPIA/PhRMA	5785	4434	76.5	0.007	0.293–0.322	$p < 0.01$
Non-industry	19 866	9074	45.7			

CI, confidence interval; EFPIA, European Federation of Pharmaceutical Industries and Associations; PhRMA, Pharmaceutical Research and Manufacturers of America; SE, standard error.

Table S4 Availability of reference to transparency in clinical trial data disclosure for non-industry institutions.

Institution	Disclosed trials, n	Total eligible trials, N	Proportion of trials disclosed, %	Reference found to transparency in clinical trial data disclosure?
**1**	394	570	69.1	Yes
**2**	353	418	84.4	No
**3**	171	339	50.4	No
**4**	112	329	34.0	No
**5**	205	318	64.5	No
**6**	149	278	53.6	No
**7**	153	259	59.1	No
**8**	88	241	36.5	No
**9**	138	241	57.3	No
**10**	65	226	28.8	No

Table S5 Exploratory analysis of the availability of results for 12 clinical trials sponsored by four biopharmaceutical companies listed in TrialsTracker

Clinical trial	Results posted in ClinicalTrials.gov	PubMed indexed in ClinicalTrials.gov	Publications found in PubMed	Scholar	Google	EU Clinical Trials Register (CTR)	Company website
**1**	No	No	No	No	Link to EU CTR summary	Yes	No
**2**	Yes	No	No	No	No	Yes	No
**3**	Yes	Yes	Yes	Link to publication	Link to CT.gov	Yes	No
**4**	Yes	Yes	Yes	Link to publication	Link to CT.gov	Yes	No
**5**	Yes	No	No	Link to publication	Link to CT.gov	No	No
**6**	Yes	Yes	Yes	Link to publication	Link to CT.gov	No	No
**7**	No	No	No	Link to publication	No	No	No
**8**	Yes	No	No	No	Link to CT.gov	No	Yes
**9**	Yes	Yes	No	Link to publication	Link to CT.gov	No	Yes
**10**	Yes	Yes	Yes	Link to publication	Link to CT.gov	Yes	Yes
**11**	No	No	No	No	No	No	Yes
**12**	Yes	No	Yes	Link to publication	Link to CT.gov	Yes	Yes

REFERENCES

1. National Institutes of Health, Department of Health and Human Services. Clinical trials registration and results information submission. Final rule. 2016. Available from https://www.ncbi.nlm.nih.gov/pubmed/27658315 (accessed 02 Februrary 2018).
2. Regulation (EU) 536/2014 of the European Parliament and of the Council of 16 April 2014 on clinical trials on medicinal products for human use, and repealing Directive 2001/20/EC. 2014. Available from https://ec.europa.eu/health/sites/health/files/files/eudralex/vol-1/reg_2014_536/reg_2014_536_en.pdf (accessed 10 April 2018).
3. European Medicines Agency. European Medicines Agency policy on publication of clinical data for medicinal products for human use. 2015. Available from http://www.ema.europa.eu/docs/en_GB/document_library/Other/2014/10/WC500174796.pdf (accessed 09 March 2018).
4. Panayi A, Baronikova S. A new age of transparency: do we fully understand the implications? International Society for Medical Publication Professionals, Inc. (ISMPP). 2017. Available from http://ismpp-newsletter.com/2017/06/29/a-new-age-of-transparency-do-we-fully-understand-the-implications/ (accessed 23 March 2018).
